# Potential serodiagnostic markers for Q fever identified in ***Coxiella burnetii ***by immunoproteomic and protein microarray approaches

**DOI:** 10.1186/1471-2180-12-35

**Published:** 2012-03-16

**Authors:** Xiaolu Xiong, Xile Wang, Bohai Wen, Stephen Graves, John Stenos

**Affiliations:** 1 State Key Laboratory of Pathogen and Biosecurity, Beijing Institute of Microbiology and Epidemiology, Dong-dia-jie, Beijing 100071, China; 2 Australian Rickettsial Reference Laboratory, Barwon Health, Geelong Hospital, Geelong, Victoria 3220, Australia

## Abstract

**Background:**

*Coxiella burnetii *is the etiological agent of Q fever. The clinical diagnosis of Q fever is mainly based on several serological tests. These tests all need Coxiella organisms which are difficult and hazardous to culture and purify.

**Results:**

An immunoproteomic study of *C. burnetii *Xinqiao strain isolated in China was conducted with the sera from experimentally infected BALB/c mice and Q fever patients. Twenty of whole proteins of Xinqiao recognized by the infection sera were identified by mass spectrometry. Nineteen of the 20 proteins were successfully expressed in *Escherichia coli *and used to fabricate a microarray which was probed with Q fever patient sera. As a result, GroEL, YbgF, RplL, Mip, OmpH, Com1, and Dnak were recognized as major seroreactive antigens. The major seroreactive proteins were fabricated in a small microarray and further analyzed with the sera of patients with rickettsial spotted fever, Legionella pneumonia or streptococcal pneumonia. In this analysis, these proteins showed fewer cross-reactions with the tested sera.

**Conclusions:**

Our results demonstrate that these 7 Coxiella proteins gave a modest sensitivity and specificity for recognizing of Q fever patient sera, suggesting that they are potential serodiagnostic markers for Q fever.

## Background

*Coxiella burnetii *is a Gram-negative bacterium that causes the worldwide zoonotic disease "Q fever". In humans, the disease generally arises from inhalation of the aerosolized Coxiella organisms produced by infected livestock. Acute Q fever usually presents as an influenza-like illness with various degrees of pneumonia [1],which may be self limiting or effectively treated with antibiotics. However, chronic Q fever is typically manifested as endocarditis, osteomyelitis or infected aortic aneurysms [1,2], and is difficult to treat.

The clinical diagnosis of Q fever is mainly based on serological tests including indirect immunofluorescence assay (IFA), enzyme-linked immunosorbent assay (ELISA) and complement fixation (CF) [1-3]. These tests have several limitations: large sample/reagent volume requirements, complex protocols, and differing sensitivities and specificities [4]. Furthermore, they all need purified Coxiella organisms which are difficult and hazardous to culture and purify [3]. Identifying novel seroreactive proteins could be a step towards the development of a fast, specific and safe molecular diagnostic assay instead of traditional serological tests. Immunoproteomic methods have been successfully applied in identifying seroreactive proteins of other pathogens [5,6]. Several immunoproteomic studies on *C. burnetii *have also been reported with various seroreactive proteins identified [7-12].

In this study, the proteins of *C. burnetii *Xinqiao, a phase I strain isolated in China [13], were analyzed with sera from experimentally infected BALB/c mice and Q fever patients using immunoproteomic analysis.

## Results

### *C. burnetii *infection in BALB/c mice

**Figure 1:**
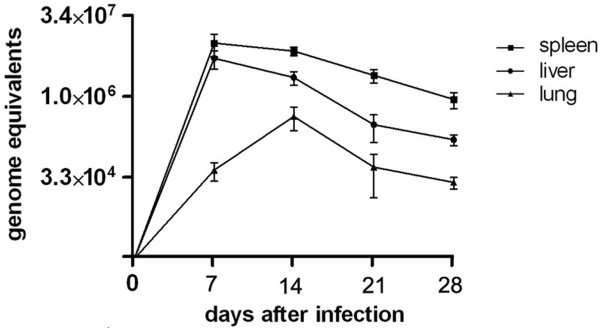
**The detection of *C. burnetii *load in BALB/c mice post-infection**. *Coxiella burnetii *load in mice organs experimentally infected and tested by real-time quantitative PCR on 0, 7, 14, 21 and 28 days pi. In quantitative PCR analysis, the copy number per mouse was obtained with 1% of the DNA sample extracted from 10 mg spleen tissue. Coxiella DNA copies were determined in groups of eight mouse samples by quantitative PCR. The results are expressed as the average copy number of eight samples on a lg scale and error bars indicate the standard deviation.

Five days post infection (pi), mice showed clinical symptoms: gathered together, reduced movement, ruffled fur, but no deaths occurred. The DNA samples extracted from tissues of the *C. burnetii*-infected mice were detected by qPCR. High levels of Coxiella DNA were found in liver and spleen tissues (Figure 1) and the highest level was found in tissues obtained on day 7 pi. The Coxiella load in spleen tissues was significantly higher than that in liver or lung tissues and significantly decreased by day 14 pi (Figure 1).

### Seroreactive proteins recognized with specific sera

**Figure 2:**
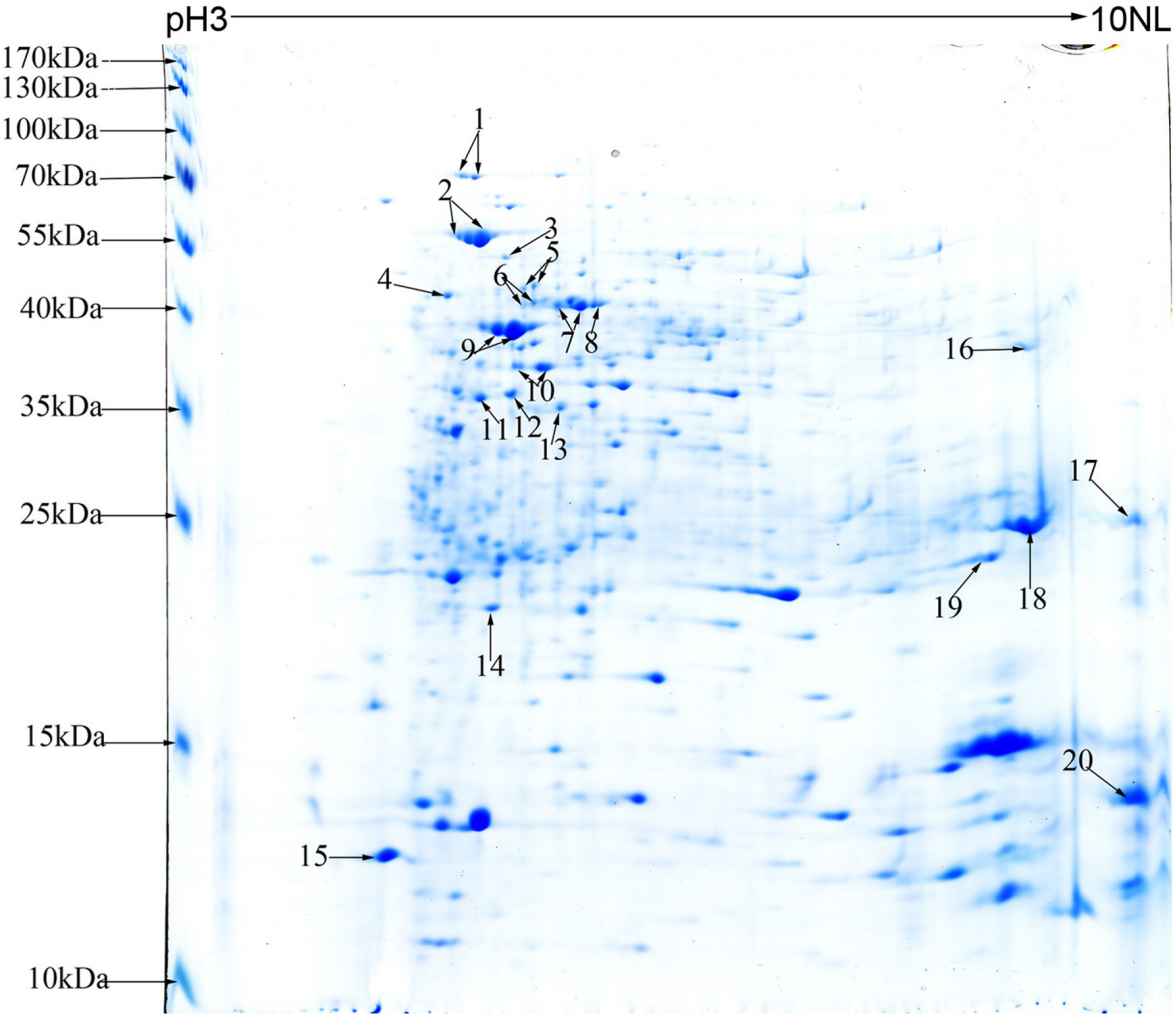
**2D gel proteome reference map of *C. burnetii *Xinqiao strain**. Isoelectric focusing was performed with a total protein extract of *C. burnetii *using a 17 cm pH 3 to 10 nonlinear Immobiline DryStrip, followed by SDS-PAGE on a 12.5% Bis-tris gel and stained by modified Coomassie brilliant blue. The numbers refer to the protein identified as shown in Table 1.

**Figure 3:**
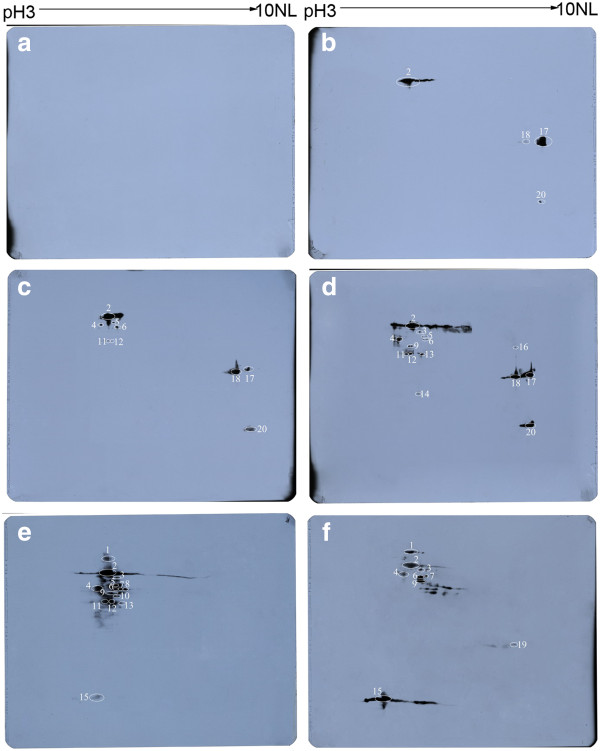
**Immunoblot analysis of the separated proteins of *C. burnetii *Xinqiao strain**. The separated proteins of *C. burnetii *Xinqiao were probed with pooled mice sera obtained at 7(A), 14(B), 21(C) and 28(D) days pi as well as two late acute Q fever patient sera (E and F), respectively. The identified antigens are denoted with circles and listed in Table 1.

The lysates of purified Coxiella organisms was separated by 2D-PAGE and a proteome map of *C. burnetii *was obtained (Figure 2). More than 500 distinct protein spots with isoelectric points (pIs) ranging from 3 to 10 and molecular mass ranging from 14 to 70 kDa were visualized by Coomassie blue stain. Following the immunoblot assay, 0, 4, 9, and 14 of the Coxiella proteins were recognized by the mice sera obtained at 7, 14, 21, and 28 days pi, respectively (Figure 3). Among these recognized proteins, 3 proteins, Chaperonin GroEL (GroEL), peptidyl-prolyl cis-trans isomerase (Mip) and putative outer membrane chaperone protein (OmpH), were strongly recognized by sera obtained at days 14, 21, and 28 days pi, and the 27 kDa outer membrane protein (Com1) was recognized by sera obtained at day 14 and strongly recognized by sera obtained on days 21 and 28 pi (Figure 3, Table 1). In addition, 15 of the Coxiella proteins were recognized by sera from two patients during the acute phase of Q fever. However, 6 of the 15 proteins, including 70 kDa chaperone protein (DnaK), LSU ribosomal protein L12P (RplL), 3-oxoacyl-[acyl-carrier-protein] synthase 2 (FabF), S-adenosylmethionine synthetase (MetK), acute disease antigen A (AdaA), glutamine synthetase (glnA), were not recognized by the mouse sera (Figure 3, Table 1).

**Table 1:** Identification of the seroreactive proteins of *C. burnetii *by MALDI-TOF-MS and ESI-MS/MS

spot no	Identification	Gene name	Locus tag	**NCBI no**.	Nominal mass	Calculated pI value	Identify method	Score	Expect value	Queries matched	%Sequence coverage	Mice sera (-days-p.i.)	Human sera(A,B)
1	Chaperone protein	*dnaK*	CBU_1290	gi|29654590	70826	5.14	MALDI-TOF	176	6.80E-12	21	38%	-	A,B
2	Chaperonin GroEL	*groEL*	CBU_1718	gi|161830449	58375	5.14	MALDI-TOF	200	2.70E-14	24	52%	14,21,28	A,B
3	Trigger factor	*tig*	CBU_0737 COXBURSA	gi|29654071	50215	5.3	MALDI-TOF	223	1.40E-16	32	67%	28	A,B
4	F0F1 ATP synthase subunit beta	*atpD*	331_A2148	gi|161830152	50490	5.01	MALDI-TOF	240	2.70E-18	26	54%	21,28	A,B
5	Dihydrolipoyllysine-residue succinyltransferase	*sucB*	CBU_1398	gi|29654691	45908	5.54	MALDI-TOF	100	0.00027	16	34%	21,28	A
6	Fructose-1,6-bisphosphate aldolase	*fbaA*	CBU_1778	gi|29655066	39793	5.41	MALDI-TOF	190	2.70E-13	16	48%	21,28	A,B
7	S-adenosylmethionine Synthetase	*metK*	CBU_2030	gi|29655311	43150	5.55	MALDI-TOF	153	1.40E-09	20	50%	-	A,B
8	3-oxoacyl-[acyl-carrier-protein] synthase 2	*fabF*	CBU_0497	gi|29653839	44275	5.49	MALDI-TOF	160	2.70E-10	20	58%	-	A
9	Elongation factor Tu	*tuf2*	CBU_0236	gi|29653588	43613	5.32	MALDI-TOF	285	8.60E-23	29	76%	28	A,B
10	Glutamine synthetase	*glnA*	CBU_0503	gi|29653845	39876	5.33	MALDI-TOF	122	1.7e-06	15	44%	-	A
11	Malate dehydrogenase	*mdh*	CBU_1241	gi|29654544	35732	5.07	MALDI-TOF	136	6.80E-08	19	50%	21,28	A
12	34 kDa outer membrane protein	*ybgF*	-	gi|30025849	33641	5.67	MALDI-TOF	92	0.0019	8	28%	21,28	A
13	(2R)-phospho-3-sulfolactate synthase	*comA*	CBU_1954	gi|29655237	33383	5.38	MALDI-TOF	146	6.80E-09	16	52%	28	A
14	Inorganic diphosphatase	*ppa*	CBU_0628	gi|29653966	19642	5.2	ESI-MS/MS	323	2.1e-26	7	36%	28	-
15	LSU ribosomal protein L12P (L7/L12)	*rplL*	CBU_0229 COXBURSA	gi|29653581	13240	4.71	ESI-MS/MS	210	4.2e-15	6	48%	-	A,B
16	30S ribosomal protein S2	*rpsB*	331_A1545	gi|161831161	35410	8.88	MALDI-TOF	100	0.00027	15	48%	28	-
17	Peptidyl-prolyl cis-trans isomerase Mip	*mip*	CBU_0630	gi|29653968	25501	9.8	MALDI-TOF	133	6.10E-07	9	57%	14,21,28	-
18	27 kDa outer membrane protein	*com1*	-	gi|11935138	26739	9.23	MALDI-TOF	95	0.00078	7	42%	14,21,28	-
19	Acute disease antigen A	*adaA*	CBU_0952	gi|29654269	25935	8.67	MALDI-TOF	110	2.70E-05	15	38%	-	B
20	Putative outer membrane Skp	*ompH*	CBU_0612	gi|29653950	18812	9.71	ESI-MS/MS	429	4.3e-37	5	28%	14,21,28	-

### Serological analysis of the recombinant seroreactive proteins with Q fever patient sera

Twenty genes encoding the seroreactive proteins were amplified (Additional file 1: Table S1) and cloned into the pET32a/pQE30 plasmid. Except for the *rpsB*-recombinant plasmid, the rest were successfully expressed in *E. coli *cells. The 19 recombinant proteins were purified by Ni-NTA agarose and analyzed by sodium dodecylsulfate polyacrylamide gel electrophoresis (SDS-PAGE). Then they were used to fabricate a protein microarray.

The protein microarray was probed with 56 sera from patients with acute Q fever and 25 sera from healthy persons (normal sera). The average FI value of the proteins probed with acute early, late or convalescent Q fever patient sera were significantly higher compared with that probed with the normal sera (*P *< 0.05) The average FI values of the proteins probed with acute late Q fever patient sera were significantly higher than acute early or convalescent Q fever patient sera (*P *< 0.05). The protein was considered to be seroreactive if its average FI probed with the patient sera were higher than the mean FI plus twice the standard deviation probed with normal sera (Additional file 2: Table S2). Seven recombinant proteins (GroEL, YbgF, RplL, Mip, Com1, OmpH, and Dnak) were selected as major seroreactive proteins with sensitivities that ranged from 48% to 88% when probed with acute late Q fever sera. The recombinant GroEL gave the highest sensitivity at 88% (Table 2).

**Table 2:** Major seroreactive proteins of *C. burnetii *on microarray probed with Q fever patient sera

	Fluorescence intensity				Sensitivity^a^		
	
Protein	Normal (n = 25)	Acute early (n = 25)	Acute late (n = 25)	Convalescent (n = 6)	Acute early	Acute late	Convalescent
GroEL	114 ± 84	1548 ± 1996	3915 ± 3462	642 ± 382	84%	88%	83%
YbgF	104 ± 83	752 ± 1308	1517 ± 1946	1176 ± 1061	44%	72%	67%
RplL	85 ± 88	277 ± 396	949 ± 1174	185 ± 119	20%	68%	17%
Mip	137 ± 78	324 ± 233	611 ± 669	237 ± 157	44%	60%	17%
Com1	70 ± 84	120 ± 326	461 ± 525	253 ± 176	12%	52%	50%
OmpH	141 ± 95	210 ± 195	676 ± 1192	398 ± 540	20%	48%	17%
DnaK	95 ± 91	143 ± 122	371 ± 480	165 ± 105	16%	48%	17%

**^a^**Sensitivity was calculated as the percentage (the number of microarray-positive sera divided by the number of sera of patients with Q fever)

### Specificity analysis of the major seroreactive proteins

A small microarray fabricated with GroEL, YbgF, RplL, Mip, Com1, OmpH, and Dnak was probed with rickettsial spotted fever, Legionella pneumonia or streptococcal pneumonia patient sera. The average FI value of each protein probed with acute late Q fever patient sera were significantly higher compared with that probed with the sera from the other three groups of patients (*P *< 0.05). A reaction was considered positive if the average FI of one protein probed with one of the tested sera were higher than the mean FI plus 2 times the standard deviation probed with the sera of healthy person sera (Additional file 3: Table S3). As a result, YbgF and DnaK displayed no reaction with any of the tested sera, and Com1 and Mip cross-reacted with one or two of the rickettsial spotted fever patient sera (Table 3). OmpH cross-reacted with one of the Legionella pneumonia or streptococcal pneumonia patient sera; GroEL cross-reacted with one of the Legionella pneumonia and two of the rickettsial spotted fever patient sera; RplL cross-reacted with two of the Legionella pneumonia and three of the streptococcal pneumonia patient sera (Table 3).

**Table 3:** Specificity analysis of the major seroreactive proteins of *C. burnetii*

	Average FI value of group sera (positive No/serum No)				
	
Protein	Q fever	Rickettsial spotted fever	Legionella pneumophila	Streptococcal pneumonia	Normal
GroEL	980 ± 1020	279 ± 60	253 ± 72	218 ± 72	207 ± 63
	(10/10)	(2/10)	(1/10)	(0/10)	(0/10)
YbgF	838 ± 442	274 ± 51	196 ± 76	197 ± 83	144 ± 111
	(10/10)	(0/10)	(0/10)	(0/10)	(0/10)
RplL	823 ± 1404	211 ± 82	251 ± 115	292 ± 188	200 ± 81
	(10/10)	(0/10)	(2/10)	(3/10)	(1/10)
Mip	361 ± 103	291 ± 31	230 ± 45	218 ± 47	227 ± 45
	(10/10)	(2/10)	(0/10)	(0/10)	(0/10)
Com1	895 ± 1145	307 ± 105	250 ± 77	226 ± 77	187 ± 105
	(10/10)	(1/10)	(0/10)	(0/10)	(0/10)
OmpH	648 ± 698	240 ± 54	217 ± 102	194 ± 142	147 ± 107
	(10/10)	(0/10)	(1/10)	(1/10)	(0/10)
DnaK	310 ± 42	226 ± 64	207 ± 66	187 ± 63	226 ± 51
	(10/10)	(0/10)	(0/10)	(0/10)	(0/10)

## Discussion

*C. burnetii *Xinqiao was isolated from ticks in China and its phase I phenotype was demonstrated in a previous study [13]. In this current study, *C. burnetii *Xinqiao was used to infect BALB/c mice and a large amount of *C. burnetii *was found in the spleens and livers of the infected mice by qPCR analysis. The Coxiella load in spleens was significantly higher compared with that in the other organs of the infected mice, indicating that the mouse spleen is the most important organ for *C. burnetii *propagation and its Coxiella load may reflect the severity of *C. burnetii *infection. The highest level of Coxiella in spleens of the infected mice was found on day 7 pi and then gradually decreased, indicating that the infected mice recovered gradually from the severe infection. These results also indicate that the combination of the sublethal challenge mouse model and the qPCR assay may be a useful and sensitive way to evaluate severity of the infection caused by different *C. burnetii *strains and evaluate efficiency of drugs or vaccines against this pathogen.

In order to identify the seroreactive proteins of *C. burnetii *Xinqiao, the whole cell lysates of the organism was separated by 2-D electrophoresis. Immunoblot analysis using the sera of mice obtained at days 14, 21, and 28 pi, indentified 4, 9, and 14 of the separated proteins, respectively. This indicated that the specific immune responses to *C. burnetii *developed progressively in the infected mice with additional antigens of *C. burentii *recognized as the immune response grew further. In addition, 15 of the proteins were recognized by sera from two patients with acute Q fever. Among these seroreactive proteins, 9 proteins were recognized by both the mouse and human sera, indicating that these proteins are able to elicit similar humoral immune responses to *C. burnetii *infection in both species.

A total of 20 seroreactive proteins were recognized by the positive mouse or human sera by mass spectra of MALDI-TOF-MS. GroEL, a conserved heat shock protein (HspB) [14], has been reported as a major immunodominant antigen of *C. burnetii *[15]. YbgF, a tol-pal system protein that involved in bacterial outer membrane stability [16], was found in both phases of *C. burnetii *[12]. GroEL and YbgF were both recognized by the sera of *C. burnetii*-infected mice and the Q fever patient sera in this study and have been previously documented as seroreactive antigens using a proteomic approach [7-9]. While Com1, Mip, and OmpH were recognized by the sera of *C. burnetii*-infected mice but were not recognized by Q fever patient sera. This difference might be due to the fact that mouse and human sera were from different infection stages or there were differences in humoral immune responses to *C. burnetii *infection between mice and humans.

Com1 was first identified as an outer membrane-associated seroreactive protein of *C. burnetii *by Hendrix and colleagues [17]. Mip is a cell-surface associated peptidylprolyl-isomerase related to macrophage infectivity potentiator protein [18] and plays a role in enhancing clearance of bacteria from spleens of infected mice [19]. OmpH is a putative outer membrane chaperone protein required for efficient release of translocated proteins from the plasma membrane [20]. The 3 proteins had also been recognized as immunodominant antigens in other studies [7,9,19,21,22]. DnaK, a surface-associated protein playing a role in assisting with folding of nascent polypeptide chains [23], and RplL, a ribosomal protein involved in translation, were previously recognized as seroreactive [9,19]. In this study, DnaK and RplL were most seroreactive when probed with the sera of patients with acute Q fever but were nonreactive when probed with the sera of *C. burnetii*-infected mice. Additionally, another 13 seroreactive proteins identified in this study were housekeeping enzymes, including FbaA, AtpD, and Tuf2 which are involved in metabolism and biosynthesis. Eight of these proteins were previously identified as seroreactive antigens [7-9,21,24]. This indicated that metabolic enzymes released from *C. burnetii *organisms were exposed to the host immune system and induced a specific antibodies response.

Nineteen of the 20 seroreactive proteins identified in this immunoproteomics study were successfully expressed in *E. coli *cells and the resultant recombinant proteins were used to fabricate a protein microarray. To evaluate their serodiagnostic potential, the protein microarray was probed with Q fever patient sera. As a result, 7 of the 19 proteins (GroEL, YbgF, RplL, Mip, Com1, OmpH, and Dnak) gave a modest sensitivity of more than 48% when probed with acute late Q fever patient sera. We noted that inconsistency existed between immunoproteomic and microarray data: the reaction of Com1 was stronger than that of Mip, OmpH or YgbF in immunoblot assay, whereas FI value of Mip, OmpH or YgbF was higher than that of Com1 in microarray assay with Q fever sera. The inconsistency might be caused by the fact that the Q fever sera recognized linear epitopes of Coxiella proteins in immunoblot assay whereas they recognized conformational epitopes of recombinant proteins in protein microarray assay.

Our results also showed that the average FI value of the 7 major seroreactive proteins probed with acute late sera were significantly higher than those probed with acute early or normal sera, which is generally in accordance with IgG titers determined in IFA. This result firmly suggests that the 7 major seroreactive proteins are immunodominant antigens of *C. burnetii *and they have capability to evoke strong humoral immune responses in *C. burnetii *infection. However, compared to IFA, the lower sensitivity of some individual proteins in microarray assay with Q fever patient sera, especially sera in acute early stage, was observed, which might be due to the fact that there were remarkable variation in immune recognition patterns for Q fever and differences between the two assays in calculating positive values. When the seroreactive proteins were analyzed in combination, 98% of antibody responders to one or more of the 7 major seroreactive proteins could be found among the Q fever patients. The remarkable variation in immune recognition patterns for Q fever requires multi-antigen combination to cover the different antibody responses and thus achieve the highest possible test sensitivity.

YbgF, RplL, Mip, Com1, and OmpH were considered as potential antigens for diagnosis of Q fever by other investigators using in vitro transcription and translation (IVTT)-based microarray of *C. burnetii *Nine Mile strain, indicated that Xinqiao strain isolated in China shares these major seroreactive antigens with Nine Mile strain [19,21]. Two heat shock proteins GroEL and Dnak were also recognized as major seroreactive antigens in this study. The positive frequencies of GroEL probed with acute early and acute late, and convalescent Q fever patient sera were 84%, 88%, and 83%, respectively, higher than the other major seroreactive proteins, suggesting that GroEL is an excellent molecular marker for Q fever. Additionally, the positive frequencies of YbgF with these Q fever patient sera were 44%, 62%, and 77%, lower than GroEL but higher than the other 5 major seroreactive proteins, indicating that it is a better protein antigen for Q fever diagnosis.

Rickettsial spotted fever caused by tick-borne infection may share similar clinical feature with Q fever. Legionella pneumonia is caused by *Legionella pneumophila *which is the bacterium closely related to *C. burnetii *with genomic homology and similar clinical presentations. Pneumonia is the major clinical presentation of acute Q fever and most bacterial pneumonia is caused by *S. pneumoniae*. These bacterial infections must be distinguished from Q fever using serological or molecular tests. Therefore, the 7 Coxiella proteins were used to fabricate a small microarray for further analysis of specificity with the sera of patients with other infectious diseases. The average FI value of each protein probed with acute late Q fever patient sera was significantly higher than that probed with the sera of patients with one of the three other infectious diseases, which indicated that the major seroreactive proteins of Coxiella can be distinguished from other bacteria in general. YbgF and DnaK displayed no cross-reaction with any of the tested sera, and Com1, Mip, OmpH and GroEL cross-reacted with one or two of the sera of patients with rickettsial spotted fever, Legionella pneumonia or bacterial pneumonia. RplL cross-reacted with two of the Legionella pneumonia patient sera and three of the streptococcal pneumonia patient sera. In this analysis, these Coxiella proteins gave a modest specificity for recognizing of Q fever patient sera, suggesting that they are potential serodiagnostic markers for Q fever.

Notably, GroEL had the highest sensitivity and modest specificity for recognizing of Q fever, which may be the most important antigen used for the diagnosis of Q fever. The antigen combination, GroEL, YbgF and Com1, may give a more authentic specificity. Refinement of antigen combination and the production of fusion molecules comprised of the major seroreactive antigens described herein may lead to improved sensitivity and specificity for the development of a rapid, accurate, and convenient seorodiagnostic test of Q fever.

## Conclusions

In summary, the combination of 2D-PAGE, immunoblot and MALDI-TOF-MS permitted the identification of 20 seroreactive proteins of *C. burnetii*. A protein microarray fabricated with recombinant proteins was probed with Q fever patient sera. Seven proteins (GroEL, YbgF, RplL, Mip, Com1, OmpH, and Dnak) were recognized as major seroreactive antigens. The major seroreactive proteins fabricated in a small array were analyzed with the sera of patients with Q fever, rickettsial spotted fever, Legionella pneumonia or streptococcal pneumonia and they gave a moderate specificity for recognizing of Q fever patient sera, suggesting these proteins are potential serodiagnostic markers for Q fever.

## Methods

### Culture and purification of *C. burnetii*

*C. burnetii *Xinqiao strain (phase I) was propagated in embryonated eggs and purified by renografin density centrifugation as previously described [25]. The purified organisms were suspended in phosphate-buffered saline buffer (PBS) (8.1 mM Na_2_HPO_4_, 1.9 mM NaH_2_PO_4_, 154 mM NaCl, PH7.4) and stored at −70°C.

### Mouse and human sera

Thirty two BALB/c mice (male, 6 weeks old) (Laboratory Animal Center of Beijing, China) were injected intraperitoneally with *C. burnetii *Xinqiao strain (1 × 10^8 ^cells/mouse) in a biosafety level 3 laboratory. Eight of the mice were randomly sacrificed on days 7, 14, 21, and 28 pi. Ten mg of tissue from the liver, spleen and lungs of each sacrificed mouse was used to extract DNA with a tissue DNA extraction kit (Qiagen, GmbH, Germany), respectively. Each DNA sample was eluted from the DNA extraction column with 200 μl elution buffer according to the manufacturer's instruction. A 2 μl of the DNA sample was tested by a real-time quantitative polymerase chain reaction (qPCR) specific for *C. burnetii *[26]. The results of qPCR were expressed as mean ± SD and compared by the repeated measurement data analysis of variance using SAS 9.1 software (SAS Institute Inc., Cary, NC). All animal protocols were pre-approved by the Animal Protection Committee of Laboratory Animal Center of Beijing and all experiments complied with the current laws of China.

Fifty six serum samples from Q fever patients were obtained from the Australian Rickettsial Reference Laboratory (Geelong, VIC, Australia) and classified into 3 types, acute early, acute late and convalescent according to the results of the IFA results and clinical details of the patients. The serum samples from 10 patients with rickettsial spotted fever and 10 patients with Legionella pneumonia caused by *Legionella pneumophila *were also obtained from the Australian Rickettsial Reference Laboratory. The serum samples of 10 patients diagnosed with streptococcal pneumonia caused by *Streptococcus pneumoniae *and 25 healthy persons were obtained from the 307 Hospital of PLA (Beijing, China). These serum samples were all Q fever antibody negative (QAb-negative) tested as described previously [27]. The present project is in compliance with the Helsinki Declaration (Ethical Principles for Medical Research Involving Human Subjects). This study was approved by the ethics committee of the Beijing Institute of Microbiology and Epidemiology. In each hospital, the serum samples of patients were collected as part of the routine management of patients without any additional sampling, and all patient data was deidentified.

### Two-dimensional (2-D) electrophoresis of *C. burnetii *proteins

The purified *C. burnetii *organisms were rinsed with cold PBS and centrifuged at 12,000 g for 30 min at 4°C with an Allegra™ 21R centrifuge (Beckman, Fullerton, CA). The supernatant was discarded and the pellet resuspended in rehydration buffer (7 M urea, 2 M thiourea, 4% [wt/vol] CHAPS, 1% [wt/vol] DTT, 0.2% [vol/vol] Bio-lyte). The cell lysates were sonicated (300 W, 3 s on and 9 s off) for 30 min at 4°C using a ultrasonic processor (Sonics & Materials, Newtown, CT), then centrifuged at 20,000 g for 1 h at 17°C to remove any insoluble material prior to isoelectric focusing. The supernatant was collected and the proteins precipitated with a 2-D Clean-Up Kit (Amersham, Piscataway, NJ) according to the manufacture's instruction. The pellets were resuspended in rehydration buffer and the protein concentration of the solution determined using the Bradford method [28]. The protein solution was aliquoted and stored at −70°C until used.

A 350 μl protein solution (800 μg of Coxiella protein) was loaded onto each 17-cm nonlinear Immobiline DryStrips (pH 3 to 10, Bio-Rad, Hercules, CA). The isoelectric focusing was performed at 50v for 12 h, 200v for 1 h, 1000v for 1 h, 10, 000v for 11 h, and 500v for 8 h using a Protean IEF cell system (Bio-Rad, Hercules, CA). Following isoelectric focusing, the strips were equilibrated and placed on sodium dodecyl sulfate (SDS)-polyacrylamide gels for second-dimension electrophoresis as described previously [29]. The gels were then stained with modified Coomassie brilliant blue [30].

### Immunoblotting of *C. burnetii *proteins

Following 2-D electrophoresis, the Coxiella proteins in the gels were transferred onto a 0.45 μm polyvinylidene difluoride membranes (Millipore, Bedford, MA) at 0.8 mA/cm^2 ^for 1 h with transfer buffer (48 mM Tris-base, 39 mM glycine, 0.04% [wt/vol] SDS, 20% [vol/vol] methanol) and then blocked overnight in blocking buffer (20 mmol/L Tris-base, 137 mmol/L NaCl supplemented with 0.05% [vol/vol] Tween 20, 5% [wt/vol] skimmed milk, pH 7.6) at 4°C. The membranes were then incubated for 1 h with mouse or patient sera diluted 1:500 in blocking buffer. After washing, the membranes were incubated for 1 h with horseradish peroxidase-conjugated goat anti-mouse or goat anti-human IgG (Santa Cruz Biotechnology, Santa Cruz, CA) diluted 1:10,000 in blocking buffer [31]. After washing, the reactivity on the membranes was detected with an ECL Western blot detection kit (Pierce, Rockford, IL).

To align Coomassie-stained gels with immunoblot images, gel images were acquired with a GS-800 calibrated imaging densitometer (Bio-Rad, Hercules, CA). The spot detection, estimation of isoelectric point (pI) and molecular weight (Mw) were done by PDQuest 2-D Analysis Software 8.0.1 (Bio-Rad, Hercules, CA). The blot images were overlaid onto parallel stained gels to allow direct comparison of spots from blot images and stained gels.

### Identification of seroreactive proteins

The Coomassie-stained protein spots that correlated with the seroreactive spots were excised and processed by Matrix-Assisted Laser Desorption/Ionization Time of Flight Mass Spectrometry (MALDI-TOF-MS). Protein digestion and MALDI-TOF-MS were performed by the National Center of Biomedical Analysis (Beijing, China). All mass spectra of MALDITOF-MS were obtained on a Bruker REFLEX III MALDI-TOF-MS (Bruker-Franzen, Bremen, Germany) as described previously [32]. The resultant peptides were mass fingerprinted and compared against the National Center for Biotechnology Information nonredundant databases using the Mascot search engine (http://www.matrixscience.co.uk). Proteins less than 20 kDa were reconfirmed by an Electrospray Ionization (ESI)-MS/MS approach and the database search was finished with a Mascot MS/MS ion search as described previously [32]. The identification process was repeated at least three times using appropriate spot candidates from different gels.

### Preparation of recombinant seroreactive proteins

The open reading frames (ORFs) of 20 seroreactive proteins recognized in the immunoproteomic assay were identified in the genome sequence of *C. burnetii *RSA 493/RSA331 (accession number NC_002971/NC_010117) with the highest sequence coverage and Mascot score. The primer pairs that amplified the 20 proteins were designed based on the DNA sequences of the ORFs(Additional file 1: Table S1)and synthesized by the Sangon Company (Sangon, Shanghai, China). Amplified gene targets were cloned into pET32a/pQE30, with the resultant recombinant proteins expressed as His (6)-tagged fusion proteins in *E. coli *BL21 (DE3)/M15 (Novagen, Madison, WI). The resultant recombinant proteins were purified by affinity chromatography with Ni-NTA resin (Qiagen, GmbH, Germany) and analysed by SDS-PAGE to test their purity and integrity according to the manufacturer's protocol.

### Fabrication of the protein microarray

The purified proteins were diluted with elution buffer (Qiagen, GmbH, Germany) to a final concentration of 200 ~ 300 μg/ml and 15 μl of each protein solution was transferred to a 384well plate and centrifuged at 300 g for 5 min in order to remove air bubbles prior to printing. The recombinant proteins were printed onto the PolymerSlide™ G slides (Captialbio, Beijing, China) using a SpotBot^® ^3 microarrayer (Arrayit corporation, Sunnyvale, CA). Five replicate spots per protein were printed, and mouse or human IgG were used as positive controls [4] and the *E. coli *cell lysate transformed with PET-32a plasmids was added as a negative control. The protein microarrays were incubated in a humid chamber at 37°C overnight and stored at 4°C. For quality control, the proteins were incubated with Cy5labeled mouse antibody (IgG) to His tag fused with the proteins on the microarray. Only the proteins with a signal-to-background ratio of ≥3.0 were used for further analysis [33].

### Serological analysis of the protein microarray

The protein microarrays were blocked in blocking buffer (8.1 mM Na2HPO4, 1.9 mM NaH_2_PO_4_, 154 mM NaCl, 1% [wt/vol] BSA, pH 7.4) for 1 h at 37°C. Human sera (1:100 dilutions) were neutralized overnight in PBS supplemented with the *E. coli *cell lysate at a final protein concentration of 5 mg/ml [21]. Fifty μl of the neutralized human sera were added to each well of the slides and incubated for 1 h at 37°C. The slides were washed 5 times with PBST (8.1 mM Na_2_HPO_4_, 1.9 mM NaH_2_PO_4_, 154 mM NaCl, 0.05% [vol/vol] Tween 20), and then incubated with Cy5-conjugated goat anti-mouse or human IgG (SBA, Gaithersburg, MD) diluted 1:500 in PBST for 1 h at 37°C. Following another 5 washes in PBST, the microarray was air dried and then scanned for fluorescent signals at a wavelength of 635 nm using a GenePix Personal 4100A scanner (Molecular Devices, Sunnyvale, CA).

The scanned images were analyzed by GenePix pro 6.0 software (Molecular Devices, Sunnyvale, CA). The fluorescence intensity (FI) of each protein was calculated by averaging the FIs of 5 replicate spots that were background subtracted. The normalized data sets were then analysed by the kruskal-wallis *H *test using SPSS 16 software (IBM, Armonk, New York).

### Specificity analysis of the major seroreactive proteins

The major seroreactive proteins identified in the above serological analysis were used to fabricate a protein microarray which was analyzed for its specificity with the sera from patients with rickettsial spotted fever, Legionella pneumonia or streptococcal pneumonia. The sera of Q fever patients and healthy persons were used as positive and negative controls, respectively. The test and data analysis method were the same as those mentioned earlier.

## Competing interests

The authors declare that they have no competing interests.

## Authors' contributions

XX carried out the experiments, data analyses and drafted the manuscript. XW assisted the analysis of microarray data; BW designed the experiments and revised the manuscript; SG and JS provided the patient sera and helped to draft the manuscript. All authors read and approved the final manuscript.

## Supplementary Material

Additional file 1 **Table S1 Primers designed for amplifying the genes encoding major seroreactive proteins**. Click here for file

Additional file 2 **Table S2 The major seroreactive proteins probed with Q fever patient sera**. Click here for file

Additional file 3 **Table S3 Specificity analysis of the major seroreactive proteins**. Click here for file
